# Bis(guanidinium) cyananilate

**DOI:** 10.1107/S1600536811028340

**Published:** 2011-08-11

**Authors:** Konstantin A. Udachin, Md. Badruz Zaman, John A. Ripmeester

**Affiliations:** a Steacie Institute for Molecular Sciences, National Research Council of Canada, 100 Sussex, Ottawa, Ontario, Canada K1A 0R6; b Center of Excellence for Research in Engineering Materials, Faculty of Engineering, King Saud University, Riyadh 11421, Saudi Arabia

## Abstract

The asymmetric unit of the title compound, 2CH_6_N_3_^+^·C_8_N_2_O_4_^2−^, contains one half of a centrosymmetric 2,5-di­cyano-3,6-dioxocyclo­hexa-1,4-diene-1,4-diolate (cyananil­ate) anion and one guanidinium cation, which are connected by N—H⋯O and N—H⋯N hydrogen bonds into a three-dimensional network.

## Related literature

For the synthesis and structure of 2,5-dihy­droxy-3,6-dicyano-1,4-benzoquinone (cyananilic acid), see: Zaman *et al.* (1996 ▶). For related cyananilic acid structures and background references, see: Zaman & Ripmeester (2010 ▶).
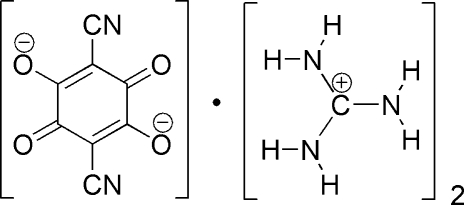


## Experimental

### 

#### Crystal data

2CH_6_N_3_^+^·C_8_N_2_O_4_^2−^
*M* *_r_* = 308.28
Monoclinic, 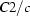

*a* = 19.4873 (17) Å
*b* = 3.6611 (3) Å
*c* = 20.2452 (18) Å
β = 112.887 (2)°
*V* = 1330.7 (2) Å^3^
*Z* = 4
Mo *K*α radiation
μ = 0.12 mm^−1^
*T* = 173 K
0.35 × 0.30 × 0.20 mm

#### Data collection

Bruker SMART 1000 CCD diffractometer
Absorption correction: multi-scan (*SADABS*; Sheldrick, 1996 ▶) *T*_min_ = 0.958, *T*_max_ = 0.976
7261 measured reflections
1704 independent reflections
1379 reflections with *I* > 2σ(*I*)
*R*_int_ = 0.026

#### Refinement

*R*[*F* ^2^ > 2σ(*F* ^2^)] = 0.044
*wR*(*F* ^2^) = 0.131
*S* = 1.08
1704 reflections
124 parameters
61 restraints
All H-atom parameters refined
Δρ_max_ = 0.44 e Å^−3^
Δρ_min_ = −0.42 e Å^−3^

### 

Data collection: *SMART* (Bruker 2003 ▶); cell refinement: *SAINT* (Bruker, 2003 ▶); data reduction: *SAINT*; program(s) used to solve structure: *SHELXS97* (Sheldrick, 2008 ▶); program(s) used to refine structure: *SHELXL97* (Sheldrick, 2008 ▶); molecular graphics: *ATOMS* (Dowty, 1999 ▶); software used to prepare material for publication: *SHELXL97*.

## Supplementary Material

Crystal structure: contains datablock(s) I, global. DOI: 10.1107/S1600536811028340/gk2374sup1.cif

Structure factors: contains datablock(s) I. DOI: 10.1107/S1600536811028340/gk2374Isup2.hkl

Supplementary material file. DOI: 10.1107/S1600536811028340/gk2374Isup3.cml

Additional supplementary materials:  crystallographic information; 3D view; checkCIF report

## Figures and Tables

**Table 1:** Hydrogen-bond geometry (Å, °)

*D*—H⋯*A*	*D*—H	H⋯*A*	*D*⋯*A*	*D*—H⋯*A*
N2—H2⋯O2^i^	0.95 (2)	2.20 (2)	3.000 (2)	142 (2)
N2—H2⋯O1^i^	0.95 (2)	2.21 (2)	3.020 (2)	143 (2)
N2—H1⋯O2	0.95 (2)	2.27 (2)	3.062 (2)	140 (2)
N3—H4⋯N1^ii^	0.92 (2)	2.14 (2)	3.025 (2)	160 (2)
N3—H3⋯O2	0.93 (2)	2.02 (2)	2.900 (2)	156 (2)
N4—H6⋯N1^ii^	0.92 (2)	2.38 (2)	3.199 (2)	148 (3)
N4—H5⋯O1^i^	0.95 (2)	1.95 (2)	2.826 (2)	151 (3)

Symmetry codes: (i) 
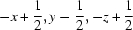
; (ii) 
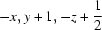
.

**Fig. 1.**
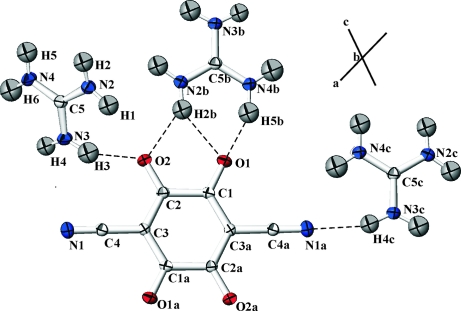
Molecular structure of the title compound with displacement ellipsoids drawn at the 50% probability level. Fragments generated by symmetry codes: (a) 1/2 - x, 1,5 - y, 1 - z; (b) 1/2 - x, 1/2 + y, 1/2 - z; (c) 1/2 + x, 2.5 - y, 1/2 + z. Hydrogen bonds are shown with dashed lines.

**Fig. 2.**
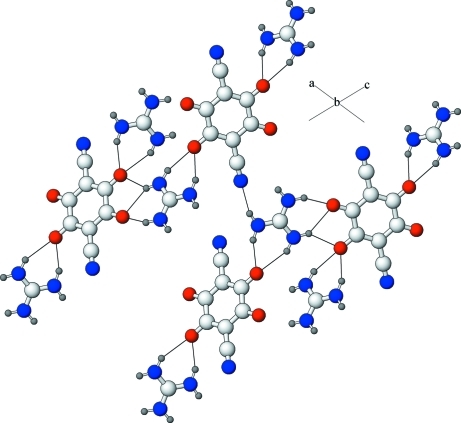
Packing diagram of the hydrogen-bonded framework structure of the title compound viewed down the b axial direction of the unit cell, showing hydrogen-bonding associations as thin lines.

## supplementary crystallographic information

### Comment

The reaction between cyananilic acid and guanidinium carbonate in methanol leads to the title compound. Since 1997, cyananilic acid (2,5-dicyano-3,6-dihydroxy-1,4-benzoquinone) has been explored due to its valuable physicochemical features. It is an organic acid that has Mott-insulator properties, and organic ferroelectricity (Zaman & Ripmeester, 2010). It forms three dimensional network through N-H···O and N-H···N hydrogen bonds (Fig. 2).

### Experimental

Cyananilic acid has been synthesized according to our published method (Zaman *et al.*, 1996) and purified by recrystallization from benzene. Light yellow compound was grown by slow evaporation of a methanol solution containing a 1:1 stoichiometric quantity of guanidinium carbonate (Aldrich, 98%) and cyananilic acid under ambient conditions. Compound decomposes at 593K.

### Refinement

N-H distances were restrained to 0.95 (2) Å and all H atoms were refined isotropically. Non- hydrogen atoms were restrained to have the same Uij components with SHELXL97 (Sheldrick, 2008) instruction 'SIMU C1 < N4'.

### Crystal data

**Table:**

2CH_6_N_3_^+^·C_8_N_2_O_4_^2^^−^	*F*(000) = 640
*M**_r_* = 308.28	*D*_x_ = 1.539 Mg m^−^^3^
Monoclinic, *C*2/*c*	Mo *K*α radiation, λ = 0.71070 Å
Hall symbol: -C 2yc	Cell parameters from 280 reflections
*a* = 19.4873 (17) Å	θ = 5.0–26°
*b* = 3.6611 (3) Å	µ = 0.12 mm^−^^1^
*c* = 20.2452 (18) Å	*T* = 173 K
β = 112.887 (2)°	Block, colourless
*V* = 1330.7 (2) Å^3^	0.35 × 0.30 × 0.20 mm
*Z* = 4	

### Data collection

**Table:**

Bruker SMART 1000 CCD diffractometer	1704 independent reflections
Radiation source: fine-focus sealed tube	1379 reflections with *I* > 2σ(*I*)
graphite	*R*_int_ = 0.026
ω scans	θ_max_ = 28.7°, θ_min_ = 2.2°
Absorption correction: multi-scan (*SADABS*; Sheldrick, 1996)	*h* = −26→26
*T*_min_ = 0.958, *T*_max_ = 0.976	*k* = −4→4
7261 measured reflections	*l* = −27→27

### Refinement

**Table:**

Refinement on *F*^2^	Primary atom site location: structure-invariant direct methods
Least-squares matrix: full	Secondary atom site location: difference Fourier map
*R*[*F*^2^ > 2σ(*F*^2^)] = 0.044	Hydrogen site location: inferred from neighbouring sites
*wR*(*F*^2^) = 0.131	All H-atom parameters refined
*S* = 1.08	*w* = 1/[σ^2^(*F*_o_^2^) + (0.078*P*)^2^ + 0.709*P*] where *P* = (*F*_o_^2^ + 2*F*_c_^2^)/3
1704 reflections	(Δ/σ)_max_ < 0.001
124 parameters	Δρ_max_ = 0.44 e Å^−^^3^
61 restraints	Δρ_min_ = −0.42 e Å^−^^3^

### Special details

**Table:**

Geometry. All e.s.d.'s (except the e.s.d. in the dihedral angle between two l.s. planes) are estimated using the full covariance matrix. The cell e.s.d.'s are taken into account individually in the estimation of e.s.d.'s in distances, angles and torsion angles; correlations between e.s.d.'s in cell parameters are only used when they are defined by crystal symmetry. An approximate (isotropic) treatment of cell e.s.d.'s is used for estimating e.s.d.'s involving l.s. planes.
Refinement. Refinement of *F*^2^ against ALL reflections. The weighted *R*-factor *wR* and goodness of fit *S* are based on *F*^2^, conventional *R*-factors *R* are based on *F*, with *F* set to zero for negative *F*^2^. The threshold expression of *F*^2^ > σ(*F*^2^) is used only for calculating *R*-factors(gt) *etc*. and is not relevant to the choice of reflections for refinement. *R*-factors based on *F*^2^ are statistically about twice as large as those based on *F*, and *R*- factors based on ALL data will be even larger.

### Fractional atomic coordinates and isotropic or equivalent isotropic displacement parameters (Å^2^)

**Table:**

	*x*	*y*	*z*	*U*_iso_*/*U*_eq_	
C1	0.30409 (9)	0.9359 (5)	0.47852 (9)	0.0199 (4)	
C2	0.23212 (9)	0.7966 (5)	0.42334 (8)	0.0194 (4)	
C3	0.18096 (9)	0.6179 (5)	0.44716 (9)	0.0193 (4)	
C4	0.11214 (10)	0.4852 (5)	0.39572 (9)	0.0213 (4)	
C5	0.11254 (10)	0.8997 (5)	0.17992 (9)	0.0221 (4)	
O1	0.34792 (8)	1.0991 (4)	0.45742 (7)	0.0288 (4)	
O2	0.22010 (7)	0.8452 (4)	0.35861 (7)	0.0254 (3)	
N1	0.05680 (9)	0.3773 (5)	0.35458 (9)	0.0297 (4)	
N2	0.17845 (9)	0.7347 (5)	0.19774 (8)	0.0255 (4)	
H2	0.1912 (16)	0.640 (8)	0.1606 (13)	0.044 (7)*	
H1	0.2039 (15)	0.661 (8)	0.2463 (11)	0.046 (7)*	
N3	0.08938 (9)	1.0023 (5)	0.23049 (9)	0.0279 (4)	
H4	0.0455 (12)	1.133 (7)	0.2154 (13)	0.039 (7)*	
H3	0.1219 (15)	0.963 (8)	0.2780 (11)	0.051 (8)*	
N4	0.07052 (10)	0.9652 (5)	0.11139 (9)	0.0294 (4)	
H6	0.0260 (13)	1.086 (8)	0.1016 (16)	0.057 (9)*	
H5	0.0886 (17)	0.897 (8)	0.0757 (14)	0.048 (8)*	

### Atomic displacement parameters (Å^2^)

**Table:**

	*U*^11^	*U*^22^	*U*^33^	*U*^12^	*U*^13^	*U*^23^
C1	0.0204 (8)	0.0214 (9)	0.0206 (8)	−0.0006 (6)	0.0110 (7)	0.0004 (6)
C2	0.0201 (8)	0.0210 (8)	0.0184 (8)	0.0008 (6)	0.0088 (6)	0.0004 (6)
C3	0.0172 (8)	0.0214 (8)	0.0192 (8)	−0.0015 (6)	0.0071 (6)	−0.0006 (6)
C4	0.0209 (8)	0.0229 (9)	0.0218 (8)	−0.0001 (7)	0.0102 (7)	0.0003 (7)
C5	0.0223 (9)	0.0237 (9)	0.0218 (8)	−0.0009 (7)	0.0103 (7)	−0.0002 (6)
O1	0.0267 (7)	0.0376 (8)	0.0259 (7)	−0.0090 (6)	0.0145 (6)	0.0012 (6)
O2	0.0250 (7)	0.0345 (8)	0.0173 (6)	−0.0013 (5)	0.0089 (5)	0.0023 (5)
N1	0.0226 (8)	0.0345 (10)	0.0293 (8)	−0.0045 (7)	0.0072 (7)	−0.0028 (7)
N2	0.0234 (8)	0.0307 (9)	0.0236 (8)	0.0043 (6)	0.0104 (6)	−0.0008 (7)
N3	0.0248 (8)	0.0394 (10)	0.0214 (8)	0.0070 (7)	0.0110 (7)	0.0005 (7)
N4	0.0261 (8)	0.0421 (10)	0.0206 (8)	0.0089 (7)	0.0099 (7)	0.0007 (7)

### Geometric parameters (Å, °)

**Table:**

C1—O1	1.246 (2)	C5—N4	1.330 (2)
C1—C3^i^	1.431 (2)	C5—N2	1.336 (2)
C1—C2	1.502 (2)	N2—H2	0.95 (2)
C2—O2	1.251 (2)	N2—H1	0.95 (2)
C2—C3	1.424 (2)	N3—H4	0.923 (19)
C3—C4	1.426 (2)	N3—H3	0.93 (2)
C4—N1	1.146 (2)	N4—H6	0.92 (2)
C5—N3	1.324 (2)	N4—H5	0.95 (2)
			
O1—C1—C3^i^	122.71 (15)	N3—C5—N2	120.05 (17)
O1—C1—C2	118.30 (15)	N4—C5—N2	120.02 (17)
C3^i^—C1—C2	118.99 (14)	C5—N2—H2	118.3 (18)
O2—C2—C3	123.33 (15)	C5—N2—H1	117.9 (17)
O2—C2—C1	118.10 (15)	H2—N2—H1	122 (2)
C3—C2—C1	118.57 (14)	C5—N3—H4	116.2 (16)
C2—C3—C4	119.52 (15)	C5—N3—H3	117.0 (18)
C2—C3—C1^i^	122.44 (14)	H4—N3—H3	126 (2)
C4—C3—C1^i^	118.04 (15)	C5—N4—H6	117.1 (19)
N1—C4—C3	179.7 (2)	C5—N4—H5	119.0 (19)
N3—C5—N4	119.93 (17)	H6—N4—H5	124 (3)

Symmetry codes: (i) −*x*+1/2, −*y*+3/2, −*z*+1.

### Hydrogen-bond geometry (Å, °)

**Table:**

*D*—H···*A*	*D*—H	H···*A*	*D*···*A*	*D*—H···*A*
N2—H2···O2^ii^	0.95 (2)	2.20 (2)	3.000 (2)	142 (2)
N2—H2···O1^ii^	0.95 (2)	2.21 (2)	3.020 (2)	143 (2)
N2—H1···O2	0.95 (2)	2.27 (2)	3.062 (2)	140 (2)
N2—H1···N2^ii^	0.95 (2)	2.64 (3)	3.319 (3)	129 (2)
N3—H4···N1^iii^	0.92 (2)	2.14 (2)	3.025 (2)	160 (2)
N3—H3···O2	0.93 (2)	2.02 (2)	2.900 (2)	156 (2)
N4—H6···N1^iii^	0.92 (2)	2.38 (2)	3.199 (2)	148 (3)
N4—H5···O1^ii^	0.95 (2)	1.95 (2)	2.826 (2)	151 (3)

Symmetry codes: (ii) −*x*+1/2, *y*−1/2, −*z*+1/2; (iii) −*x*, *y*+1, −*z*+1/2.
